# An Enquiry, as to Whether and How “Enemata Nutrientiæ” Are Absorbed from the Rectum, so as to Prove Nutrient to the System

**Published:** 1859-02

**Authors:** B. Woodward

**Affiliations:** Galesburg, Ill.


ARTICLE III.

### AN ENQUIRY, AS TO WHETHER AND HOW "ENEMATA NUTRIENTIÆ" ARE ABSORBED FROM THE RECTUM, SO AS TO PROVE NUTRIENT TO THE SYSTEM.

BY B. WOODWARD, M.D., OF GALESBURG, ILL.

In a late work by Wm. Tully, M.D., on "Materia Medicæ, or Pharmacology," he denies the efficacy of nutrient enemata, and says, "The colon and rectum having no lacteals, the idea of introducing nourishment by them is a perfect chimera."

It sometimes happens that very learned men make very learned mistakes, and it seems to me that this is one of them. The lacteals are, no doubt, the main organs by which the nutrient particles of food are taken up and carried into the circulation, yet there are others acting consentaneously with the lacteals for the attainment of the same end. Though the villi containing the lacteals do not extend below the cæcum, and these lacteals are the *main* organs of absorption from the intestinal canal, there are still the capillary bloodvessels, which, according to our best physiologists, are "equally with the lacteals actively engaged in the process of absorption." See "Carpenter's Human Physiology," p. 134.

How will the Doctor dispose of this theory, sustained as it is by facts? He says absorption cannot be carried on because there are no vessels suited to the process. Had he said that *digestion* could not be carried on in the rectum and colon, I should have been more inclined to agree with him, for digestion is the conversion of food into chyle, and its subsequent absorption; though even in this case it is not certain that the presence of food in the duodenum is necessary for the pancreas to secrete its fluid, any more than it is necessary for the liver, though this condition does seem to be necessary in the case of the stomach; and if these secretions (which seems to me highly probable) are carried into the colon and rectum, they may there so act on nutrient enema as to convert them into chyle and fit them to be taken up by the capillary vessels. It is generally believed that starchy and farinaceous matters need to undergo this process of digestion, though Carpenter, p. 145, holds that this change is not necessary even for these articles, in order to their absorption. In the case of animal broths, this conversion certainly is not necessary, for the constituents of the flesh are held in perfect solution in the water, leaving little else than what would be excrement in the ordinary process of digestion, to be retained in the intestine. The rest, the gelatine, fibrine, salts, and perhaps even the albumen, are in a fit condition to be taken up by the capillaries of the part. Even granting that they could not be absorbed from the rectum and colon, we have reason to believe, that being deposited in a fluid form, they may be carried by capillary attraction along the walls of the large into the small intestines, and there undergo the change into chyle and be absorbed by the lacteals. It may be said that "the lacteals have open mouths, fitting them for absorbing, which the capillary vessels have not." True, but this is not necessary to maintain the process. It is carried on by endosmose through the walls of the vessels themselves, and the greater part of the nutrient particles held in solution are taken up and carried at once into the circulation. The influence of the capillary vessels is seen even in the skin, and still more so in an abraded surface when medicinal substances are applied to these surfaces; the fact of their absorption being proved by finding their effects upon the system after having been thus applied. That nutrient enemata are absorbed from the rectum, is shown by the fact, that after a certain time they are neither found in the gut nor passed as excrement. With regard to medicinal substances, they are proved to be absorbed from the rectum, by finding not only their appropriate constitutional effects, but also their presence in the urine, which could not be, unless they had fully entered the circulation. We might reason from analogy that the rectum might absorb, by finding, as we do, other organs which are only excretory in their nature, taking on, under certain circumstances, the *absorbent* action. The urethra is one case in point; for it is well known that the most intense narcotism may be produced by the introduction of morphine into the canal. This could only take place through the action of the capillary vessels of the part; and though the action of this agent, as well as many others, is on the nervous system, it must enter the circulation before it can affect the nervous centres. We believe that menstruation is but a consequence of ovulation, and though the uterus and vagina is the natural course for this secretion, we still find various other parts of the system at times taking on a vicarious action, and for want of action of the uterus, doing its excretory work. If the theory of Dr. Tully is true, how are we to account for the continuation of life, and subsequent recovery of the patient in those cases where food has been introduced in no other way than by enema, for such periods as from ten to seventeen days? and yet such cases are frequently occurring. That it requires a much larger quantity of food introduced in this way, I admit, in order for sustentation to be carried on; but must be allowed to doubt Dr. Tully's theory. Dr. Tully's work will doubtless fall into the hands of many young and inexperienced practitioners. If they adopt his views on this subject, they will refrain from using this (what most physicians find to be a most efficacious) means of supporting their patients in cases where from any cause food cannot be retained on the stomach.

Fully aware that it may look like presumption to endeavor to controvert the views of distinguished men, yet we are bound to exercise our own judgment, based on the best knowledge we can acquire. Dr. Tully claims for himself the right to reject any opinion or theory, no matter from what source it may come, if it does not coincide with his views of truth; and the same liberty must be granted to every member of the profession, or there can be no advance in medical science. Dr. Tully says, very truly, "That an assertion once made can never be got rid of. The writer who first made the assertion will be quoted till doomsday; no correction of such an error can ever rid the profession of it." This is so true that it becomes the duty of every man to examine every question for himself, and take nothing for granted because it emanates from a distinguished source. The Doctor controverts, in this case, what he believes to be a "hoary error," but what, if I mistake not, the profession generally believe to be a "time-honored truth," that the system may be effectively supported by "Enemata Nutrientiæ."

